# Lymph node ratio as a prognostic factor in head and neck cancer patients

**DOI:** 10.1186/s13014-015-0490-9

**Published:** 2015-08-25

**Authors:** Chien-Chih Chen, Jin-Ching Lin, Kuan-Wen Chen

**Affiliations:** Department of Radiation Oncology, Taichung Veterans General Hospital, No.1650, Sect. 4, Taiwan Boulevard, Taichung, 40705 Taiwan Republic of China; Department of Medicine, School of Medicine, National Yang-Ming University, Taipei City, Taiwan Republic of China

## Abstract

**Background:**

Lymph node status is one prognostic factor in head and neck cancer. The purpose of this study is to investigate the prognostic value of lymph node ratio (LNR) in head and neck cancer patients who received surgery plus postoperative chemoradiotherapy.

**Methods:**

From May 1991 to December 2012, a total of 117 head and neck cancer patients who received surgery plus postoperative chemoradiotherapy were analyzed. The primary sites were oral cavity (93), oropharynx (13), hypopharynx (6), and larynx (5). All patients had pathologically confirmed squamous cell carcinoma and 63 patients had neck lymph nodes metastasis. LNR was calculated for each patient. The endpoints were overall survival (OS), local failure-free survival (LFFS), and distant metastasis-free survival (DMFS).

**Results:**

The median follow up time was 36 months, with a range from 3.4 to 222 months. The 3-year rates of OS, LFFS, and DMFS were 59.7, 70.3, and 81.8 %, respectively. The median value of LNR for lymph nodes positive patients was 0.1. In univariate analysis, patients with an LNR value less than 0.1 had better 3-year OS (67.0 % vs.41.0 %, *p* = 0.004), 3-year LFFS (76.1 % vs. 54.9 %, *p* = 0.015) and 3-year DMFS (87.2 % vs. 66.4 %, *p* = 0.06).

Multivariate analysis revealed that LNR was an independent prognostic factor for OS (hazard ratio [HR] = 2.92; 95 % confidence interval [CI] = 1.367–6.242; *p* = 0.006) and LFFS (HR = 4.12; 95 % CI = 1.604–10.59; *p* = 0.003).

**Conclusion:**

LNR is an important prognosis factor for OS and LFFS in head and neck cancer patients.

**Electronic supplementary material:**

The online version of this article (doi:10.1186/s13014-015-0490-9) contains supplementary material, which is available to authorized users.

## Background

Squamous cell carcinoma (SCC) of head and neck is one of the common malignant tumors worldwide. The mainstay of treatment is surgery; surgery plus chemotherapy and/or radiotherapy are used for treatment of advanced disease [1–3]. However, even after patients receive surgery plus adjuvant therapy for head and neck cancer, some of them may still experience relapse. Therefore, it is important to improve treatment outcome by finding reliable prognostic factors and identifying head and neck cancer patients at high risk of relapse.

One of the most commonly used prognostic factors is the tumor-node-metastasis (TNM) staging system. The TNM staging system classifies lymph nodes status by the number, size, and laterality of positive lymph nodes [4]. However pathologic lymph node status and current nodal classification may not necessarily predict prognosis [5].

Lymph nodes ratio (LNR), defined as the ratio of the number of positive lymph nodes to the total number of lymph nodes removed, is used as a prognostic factor in patients with bladder cancer [6, 7], esophageal cancer [8] and cervical cancer [9]. Some studies [10–13] showed that LNR could predict the clinical outcomes in head and neck cancer patients.

The purpose of this study was to investigate the prognostic value of LNR in head and neck cancer patients who received surgery plus postoperative chemoradiotherapy.

## Materials and methods

### Patients

We reviewed the database of patients who were newly diagnosed with head and neck cancer from May 1991 to December 2012 at Taichung Veteran General Hospital. The inclusion criteria were patients: (1) who underwent a complete pretreatment staging workup, and had no distant metastasis at diagnosis; (2) with pathologically confirmed SCC; (3) who received radical tumor excision with adequate margin and neck dissection; and (4) who received adjuvant chemoradiotherapy. There were 117 eligible patients in this cohort study. Pathologic lymph node status was evaluated by 2 pathologists and LNR was calculated for each patient. The final staging was done according to the American Joint Committee on Cancer (AJCC) TNM classification system 7^th^ edition. The study was approved by the Institutional Review Board of Taichung Veterans General Hospital.

### Chemoradiotherapy

All patients were scheduled to undergo external beam radiotherapy using a linear accelerator with a 6-MV photon beam and source-axis distance technique. A total radiation dose of 60.0–73.8 Gy, 1.8–2.0 Gy per fraction, 5 fractions per week was delivered. A radiation does of 60–70 Gy was used in all patients except one, who received 73.8 Gy.

Concurrent chemotherapy consisted of cisplatin 20 mg/m^2^ and 5FU 400 mg/m^2^ for 1–4 days, during the first and fifth week of radiotherapy, or weekly cisplatin 30–50 mg/m^2^.

### Statistical analysis

The endpoints were overall survival (OS), local failure-free survival (LFFS) and distant metastasis-free survival (DMFS). The OS was calculated from the date of surgery to the date of death from any cause or last follow-up. The LFFS was measured from the date of surgery to the date of any evidence of local recurrence or last follow-up. The DMFS was calculated from the date of surgery to the date of distant metastasis or last follow-up. We analyzed the impact of LNR on OS, LFFS, and DMFS. Survival times were estimated using the Kaplan-Meier method and Log-rank test was used for the comparison between the groups. A Cox regression model was used for multivariate analysis. The statistical analyses were performed using SPSS software, version 10.0. A p value less than 0.05 was considered statistically significant.

## Results

Table 1 summarizes the patients’ characteristics. There were 110 males and 7 females. The median age was 51 years, with a range from 34–74 years. The primary site included the oral cavity (93), oropharynx (13), hypopharynx (6), and larynx (5). Histopathologic examination revealed that 35 patients had positive margin, 63 patients had lymph nodes metastasis, and 25 patients had extracapsular extension.

**Table 1:** Patients characteristic (*n* = 117)

		Number
Age	34–74	
	Median: 51	
Stage	I	4
	II	15
	III	14
	IV	84
Margin	Positive	35
	Negative	82
Lymph nodes status	Positive	63
	Negative	54
Extracapsular extension	Positive	25
	Negative	92
Lymph nodes ratio	<0.1	84
	≧0.1	33

Additional file 1: Table S1 showed the absolute number of lymph nodes metastases and LNR distribution of all patients. The absolute number of metastatic lymph node ranged from 1 to 15. Twenty patients had single lymph node metastasis and 43 patients had 2 or more lymph nodes metastases. The median value of LNR for lymph node-positive patients was 0.1, with a range from 0.01 to 1.0. We categorized all patients into LNR ≧ 0.1 group and LNR < 0.1 group. Patients with negative lymph nodes were assigned to the LNR < 0.1 group. There were 84 patients in the LNR < 0.1 group, and 33 patients in the LNR ≧ 0.1 group. For 63 lymph node-positive patients, we divided these patients into high (LNR > 0.17), medium (LNR: 0.06–0.17) and low (LNR < 0.06) LNR group.

**Fig. 1:**
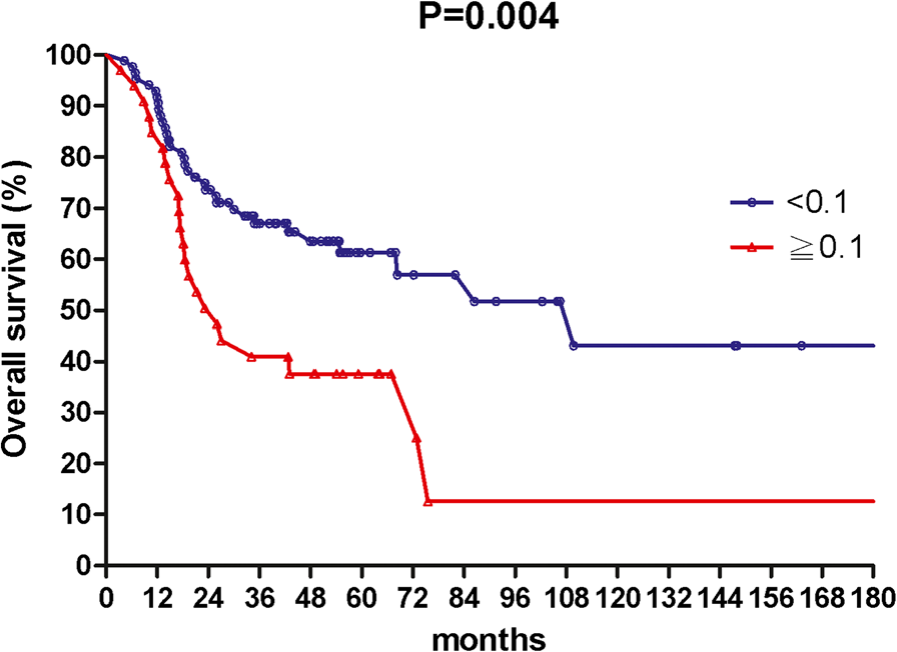
Overall survival according to lymph nodes ratio for all patients

**Fig. 2:**
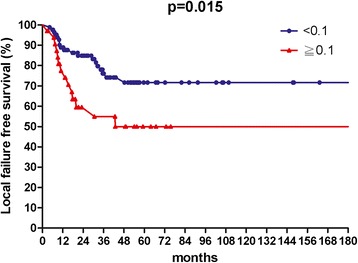
Local failure free survival according to lymph nodes ratio for all patients

**Fig. 3:**
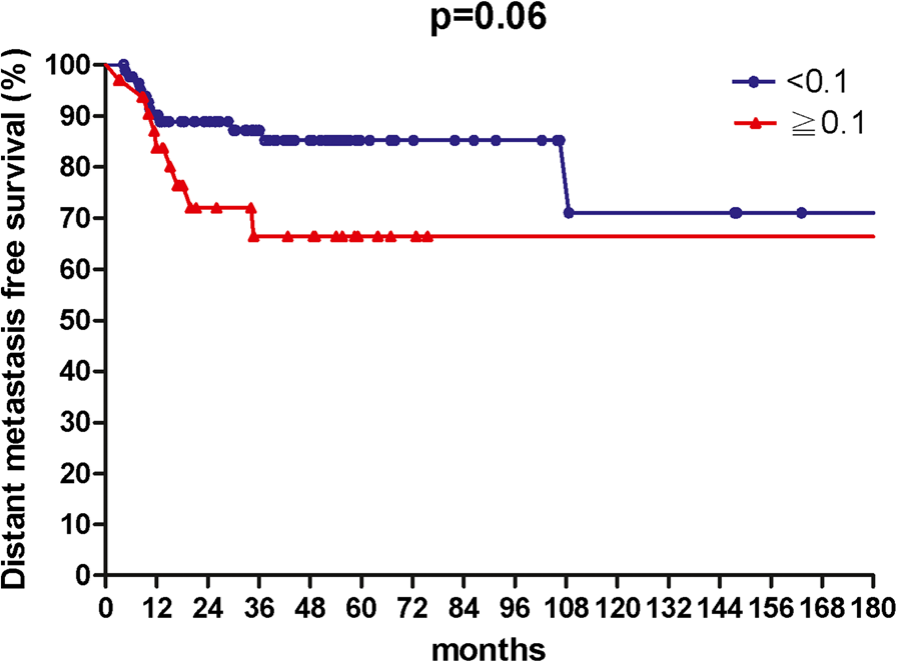
Distant metastasis free survival according to lymph nodes ratio for all patients

**Fig. 4:**
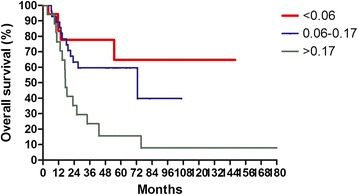
Overall survival according to lymph nodes ratio for 63 lymph node-positive patients

**Fig. 5:**
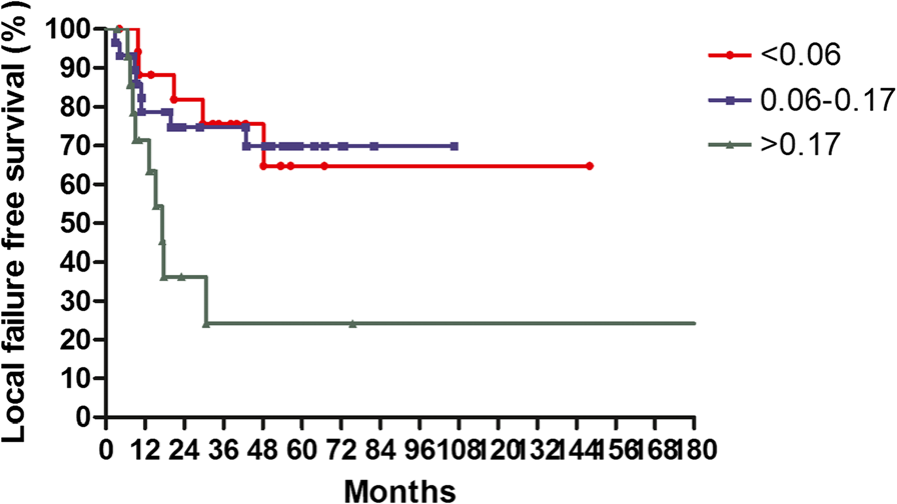
Local failure free survival according to lymph nodes ratio for 63 lymph node-positive patients

**Fig. 6:**
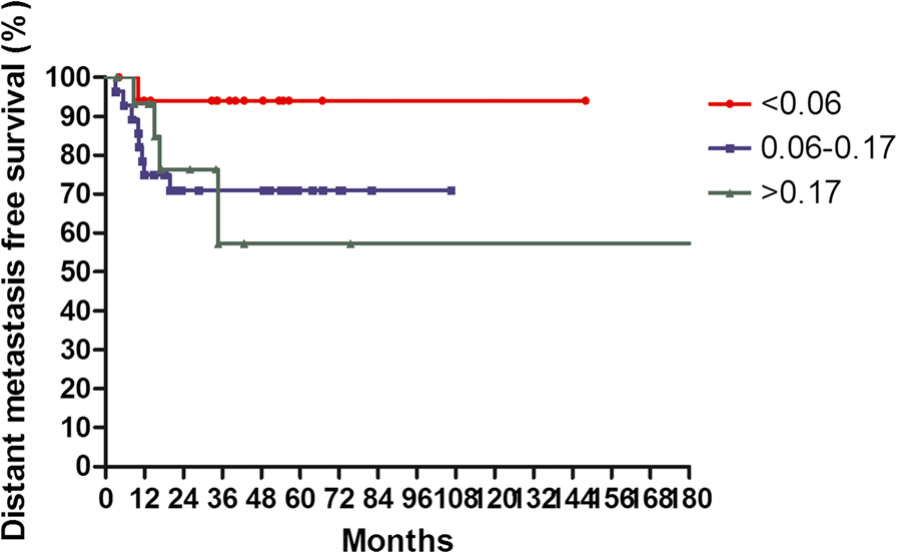
Distant metastasis free survival according to lymph nodes ratio for 63 lymph node-positive patients

The median follow-up time was 36 months, with a range from 3.4 to 222 months. For surviving patients, the follow up time was at least 2 years. The 3-year OS, LFFS, and DMFS for all patients were 59.7, 70.3, and 81.8 %, respectively. There were no treatment-related death in this study. Table 2 summarizes the results of univariate analysis. Patients with LNR <0.1 had longer 3-year OS (67.0 % vs.41.0 %, *p* = 0.004, Fig. 1). Patients with LNR <0.1 had higher 3-year LFFS (76.1 % vs. 54.9 %, *p* = 0.015, Fig. 2). Patients with LNR <0.1 tended to have better 3-year DMFS (87.2 % vs. 66.4 %, *p* = 0.06, Fig. 3). The subgroup analysis for lymph node-positive patients showed that higher LNR had poor 3-year OS (*p* = 0.003, Fig. 4) and LFFS (*p* = 0.011, Fig. 5). High LNR group had poorer DMFS than medium and low LNR group (Fig. 6), but it did not reached statistically significance (*p* = 0.18). The absolute number of lymph node metastases had no significant difference on OS (*p* = 0.28), LFFS (*p* = 0.46) and DMFS (*p* = 0.96).

**Table 2:** Univariate analysis for three- year LFFS, DMFS, and OS

Parameter	LFFS (%)	DMFS (%)	OS (%)
Age			
≦51	61.3	76.1	58.4
> 51	79.9	88.0	63.4
	*p* = 0.02	*p* = 0.13	*p* = 0.76
Tumor site			
Oral cavity	64.8	81.7	56.4
others	88.0	82.9	70.4
	*p* = 0.06	*p* = 0.46	*p* = 0.28
Stage			
I + II	80.2	100	71.5
III + IV	68.3	76.6	57.4
	*p* = 0.23	*p* = 0.04	*p* = 0.13
Margin			
Positive	64.6	89.5	41.0
Negative	73.3	79.2	67.6
	*p* = 0.85	*p* = 0.14	*p* = 0.05
Extracapsular extension			
Positive	68.7	77.0	64.0
Negative	70.8	83.4	58.6
	*p* = 0.76	*p* = 0.93	*p* = 0.11
LNR for all patients			
< 0.1	76.1	87.2	67.0
≧0.1	54.9	66.4	41.0
	*p* = 0.015	*p* = 0.06	*p* = 0.004
LNR for lymph node-positive patients			
< 0.06	75.6	94.1	77.8
0.06–0.17	74.8	71.1	59.7
> 0.17	24.2	57.3	23.5
	*p* = 0.011	*p* = 0.18	*p* = 0.003
Absolute number of lymph node metastasis			
1	60.0	75.8	48.5
> 1	66.4	76.6	58.0
	*p* = 0.46	*p* = 0.96	*p* = 0.28

*OS* overall survival, *LFFS* local failure-free survival, *DMFS* distant metastasis-free survival, *LNR* lymph node ratio

To investigate whether LNR was an independent prognostic factor for OS, LFFS and DMFS for head and neck patients, we conducted a multivariate analysis with relevant variables. Table 3 summarizes the results of the multivariate analysis. LNR was an independent prognostic factor for OS (hazard ratio [HR] = 2.92; 95 % confidence interval [CI] = 1.367–6.242; *p* = 0.006) and LFFS (HR = 4.12; 95 % CI = 1.604–10.59; *p* = 0.003). LNR also showed borderline statistically difference on DMFS (HR = 3.10; 95 % CI = 0.957–10.09; *p* = 0.059).

**Table 3:** Multivariate analysis for OS, LFFS, DMFS

Variables	HR (95 % CI)	*P* value
OS		
Age (>51 vs. ≦51)	0.77 (0.440–1.342)	0.354
Gender (male vs. female)	4.11 (0.942–17.92)	0.060
ECE (negative vs. positive)	0.55 (0.257–1.163)	0.117
Margin (negative vs. positive)	0.52 (0.295–0.916)	0.024
LNR (≧0.1 vs. < 0.1)	2.92 (1.367–6.242)	0.006
Absolute number of lymph nodes metastases (1 vs. ≧2)	1.02 (0.661–1.575)	0.928
Stage (III + IV vs I + II)	1.99 (0.744–5.319)	0.170
Primary (other primary site vs. oral cavity)	0.59 (0.303–1.160)	0.127
LFFS		
Age (>51 vs. ≦51)	0.34 (0.156–0.729)	0.006
Gender (male vs. female)	6.10 (0.806–46.14)	0.080
ECE (negative vs. positive)	0.39 (0.145–1.040)	0.060
Margin (negative vs. positive)	0.81 (0.358–1.837)	0.615
LNR (≧0.1 vs. < 0.1)	4.12 (1.604–10.59)	0.003
Absolute number of lymph nodes metastases (1 vs. ≧2)	1.15 (0.674–1.967)	0.606
Stage (III + IV vs I + II)	2.09 (0.576–7.567)	0.263
Primary (other primary site vs. oral cavity)	0.33 (0.112–0.957)	0.041
DMFS		
Age (>51 vs. ≦51)	0.34 (0.132–0.865)	0.024
Gender (male vs. female)	2.80 (0.360–22.14)	0.323
ECE (negative vs. positive)	0.68 (0.225–2.057)	0.495
Margin (negative vs. positive)	2.37 (0.682–8.256)	0.175
LNR (≧0.1 vs. < 0.1)	3.10 (0.957–10.09)	0.059
Absolute number of lymph nodes metastases (1 vs. ≧2)	0.85 (0.444–1.635)	0.630
Stage (III + IV vs I + II)	6.90 (0.176–41.03)	0.199
Primary (other primary site vs. oral cavity)	0.74 (0.238–2.270)	0.592

*OS* overall survival, *LFFS* local failure free survival, *DMFS* distant metastasis-free survival

## Discussion

Surgery is the mainstay treatment for head and neck cancer, and the standard operation for head and neck cancer patients is resection of primary tumor with adequate margins for local control and neck dissection for neck control. One of the most significant prognostic factors is neck lymph nodes metastasis [14]. In the study by Mamalle et al. [14], the number of positive lymph nodes was found to be a predictor of outcome for head and neck cancer patients. Previous studies [15, 16] showed that a significantly likelihood of finding neck lymph nodes metastasis was associated with increased in the total number of dissected lymph nodes. But Gil et al. [10] found no significant correlation between the total number of excised lymph nodes (positive and negative) and the number of positive lymph nodes in the specimen. The number of positive lymph nodes and the total number of dissected lymph nodes are affected by a lymph nodes dissection procedure and confirmed by pathological examination. The LNR may have a higher prognostic value in determining lymph nodes status, because disease (the number of positive lymph nodes), treatment option (neck dissection procedure and total number of dissected lymph nodes) and the diagnosis of a pathologist are considered at the same time.

To our knowledge, few studies analyzed LNR in patients with different head and neck cancer. Gil et al. [10] analyzed 386 oral cavity cancer patients who received primary surgery with or without adjuvant radiotherapy and showed LNR remained the only independent predictor of OS (HR = 2.0, *p* = 0.02), disease specific survival (DSS) (HR = 2.3, *p* = 0.02), and local control (HR = 4.1, *p* = 0.005). Kim et al. [11] analyzed 211 oral cavity cancer patients who underwent surgery and found that LNR was an independent predictor of DSS (HR = 3.24, 95 % CI = 1.61–6.53; *p* = 0.001). In our study, we found LNR was an independent prognostic factor for OS (HR = 5.14; 95 % CI = 2.026–13.07; *p* = 0.001) and LFFS (HR = 12.60; 95 % CI = 3.872–37.5; *p* < 0.001) for head and neck cancer patients who received surgery and adjuvant chemoradiotherapy.

The cutoff value for LNR varied across studies. Gil et al. [10] used a cutoff value of 0.06. Sayed et al. [13] reviewed medical data of 1408 oral cancer patients and found LNR (0.088) was significantly associated with survival outcomes. Hua et al. [12] analyzed 81 hypopharyngeal cancer patients and revealed that those with an LNR ≧ 0.1 had poor OS. In our study, we used a cutoff value of 0.1 to categorize patients into LNR ≧ 0.1 group and LNR < 0.1 groups. Shrime et al. [17] categorized oral SCC patients into low (0–6 %), moderate (6–13 %) and high (>13 %) risk groups based on nodal ratio. Ebrahimi et al. [18] used multiple LNR cutoff points to analyze survival outcomes of oral cancer patients who received surgery and discovered that LNRs are important in prognostic models for node‐positive patients. Previous studies did not define a specific cutoff value for LNR, but patients with a higher LNR were shown to have poor survival.

This retrospective study had some limitations. First, different primary sites and different operation procedures may have affected the treatment results. Second, the total number of dissected lymph nodes depended on disease and physicians’ lymph node criteria, this may have affected the LNR results. The main advantage of this study was that all patients received surgery plus adjuvant chemoradiotherapy. Compared to previous study, our treatment was more consistent. Consistent adjuvant therapy can reduce the effects of different treatments. Further study is needed to confirm the prognostic value of LNR in head and neck cancer patients.

## Conclusion

LNR is an independent prognosis factor for OS and LFFS in head and neck cancer patients. In addition to the AJCC TNM classification system, LNR may be useful in stratifying risk in patients with head and neck cancer.

## Additional file

Additional file 1: Table S1. The LNR distribution of all patients. (DOC 221 kb)

## Abbreviations

LNR: Lymph node ratio
OS: Overall survival
LFFS: Local failure-free survival
DMFS: Distant metastasis-free survival
SCC: Squamous cell carcinoma
TNM staging system: Tumor-node-metastasis staging system
AJCC: American Joint Committee on Cancer
DSS: Disease specific survival
