# Supplementary material for: Lymph node ratio as a prognostic factor in head and neck cancer patients
Source: Radiat Oncol. 2015 Aug 25;10:181. doi: 10.1186/s13014-015-0490-9 (PMC4554293; doi:10.1186/s13014-015-0490-9)
Supplement: Additional file 1: Table S1. — The LNR distribution of all patients. (DOC 221 kb) [file 13014_2015_490_MOESM1_ESM.doc]

| Supplementary table 1 | | | | | | | | |
| --- | --- | --- | --- | --- | --- | --- | --- | --- |
| SEX | AGE | T | N | M | SITE | LN positive  number | Total dissected LNs | LNR |
| M | 51 | 4 | 0 | 0 | hypopharynx | 0 | 8 | 0.00 |
| M | 53 | 4 | 0 | 0 | larynx | 0 | 29 | 0.00 |
| M | 47 | 4 | 0 | 0 | oral cavity | 0 | 43 | 0.00 |
| M | 34 | 4 | 0 | 0 | oral cavity | 0 | 6 | 0.00 |
| M | 48 | 4 | 0 | 0 | oral cavity | 0 | 33 | 0.00 |
| M | 54 | 4 | 0 | 0 | oral cavity | 0 | 5 | 0.00 |
| M | 49 | 2 | 0 | 0 | oral cavity | 0 | 7 | 0.00 |
| M | 42 | 4 | 0 | 0 | oral cavity | 0 | 9 | 0.00 |
| M | 65 | 4 | 0 | 0 | oral cavity | 0 | 38 | 0.00 |
| M | 40 | 2 | 0 | 0 | oral cavity | 0 | 14 | 0.00 |
| M | 48 | 2 | 0 | 0 | oral cavity | 0 | 11 | 0.00 |
| M | 36 | 4 | 0 | 0 | oral cavity | 0 | 33 | 0.00 |
| M | 35 | 4 | 0 | 0 | oral cavity | 0 | 25 | 0.00 |
| M | 44 | 2 | 0 | 0 | oral cavity | 0 | 51 | 0.00 |
| M | 58 | 2 | 0 | 0 | oral cavity | 0 | 32 | 0.00 |
| F | 39 | 4 | 0 | 0 | oral cavity | 0 | 20 | 0.00 |
| M | 45 | 2 | 0 | 0 | oral cavity | 0 | 19 | 0.00 |
| M | 42 | 4 | 0 | 0 | oral cavity | 0 | 14 | 0.00 |
| M | 47 | 3 | 0 | 0 | oral cavity | 0 | 27 | 0.00 |
| M | 56 | 2 | 0 | 0 | oral cavity | 0 | 4 | 0.00 |
| M | 49 | 1 | 0 | 0 | oral cavity | 0 | 11 | 0.00 |
| M | 39 | 4 | 0 | 0 | oral cavity | 0 | 58 | 0.00 |
| M | 60 | 4 | 0 | 0 | oral cavity | 0 | 18 | 0.00 |
| M | 47 | 3 | 0 | 0 | oral cavity | 0 | 36 | 0.00 |
| M | 34 | 1 | 0 | 0 | oral cavity | 0 | 12 | 0.00 |
| M | 57 | 4 | 0 | 0 | oral cavity | 0 | 22 | 0.00 |
| M | 43 | 2 | 0 | 0 | oral cavity | 0 | 12 | 0.00 |
| M | 61 | 2 | 0 | 0 | oral cavity | 0 | 25 | 0.00 |
| M | 58 | 2 | 0 | 0 | oral cavity | 0 | 48 | 0.00 |
| M | 63 | 4 | 0 | 0 | oral cavity | 0 | 54 | 0.00 |
| M | 67 | 2 | 0 | 0 | oral cavity | 0 | 21 | 0.00 |
| M | 42 | 4 | 0 | 0 | oral cavity | 0 | 94 | 0.00 |
| M | 56 | 4 | 0 | 0 | oral cavity | 0 | 30 | 0.00 |
| M | 55 | 4 | 0 | 0 | oral cavity | 0 | 47 | 0.00 |
| M | 47 | 1 | 0 | 0 | oral cavity | 0 | 13 | 0.00 |
| M | 62 | 4 | 0 | 0 | oral cavity | 0 | 18 | 0.00 |
| M | 39 | 4 | 0 | 0 | oral cavity | 0 | 11 | 0.00 |
| M | 48 | 2 | 0 | 0 | oral cavity | 0 | 12 | 0.00 |
| M | 68 | 4 | 0 | 0 | oral cavity | 0 | 33 | 0.00 |
| M | 54 | 4 | 0 | 0 | oral cavity | 0 | 27 | 0.00 |
| M | 71 | 4 | 0 | 0 | oral cavity | 0 | 8 | 0.00 |
| M | 44 | 4 | 0 | 0 | oral cavity | 0 | 13 | 0.00 |
| M | 47 | 4 | 0 | 0 | oral cavity | 0 | 17 | 0.00 |
| M | 48 | 4 | 0 | 0 | oral cavity | 0 | 16 | 0.00 |
| M | 43 | 4 | 0 | 0 | oral cavity | 0 | 9 | 0.00 |
| M | 48 | 4 | 0 | 0 | oral cavity | 0 | 12 | 0.00 |
| M | 52 | 4 | 0 | 0 | oral cavity | 0 | 19 | 0.00 |
| M | 63 | 2 | 0 | 0 | oral cavity | 0 | 14 | 0.00 |
| M | 54 | 4 | 0 | 0 | oropharynx | 0 | 54 | 0.00 |
| M | 56 | 2 | 0 | 0 | oropharynx | 0 | 14 | 0.00 |
| M | 72 | 4 | 0 | 0 | oropharynx | 0 | 21 | 0.00 |
| M | 60 | 2 | 0 | 0 | oropharynx | 0 | 46 | 0.00 |
| M | 49 | 1 | 0 | 0 | oropharynx | 0 | 17 | 0.00 |
| M | 51 | 4 | 0 | 0 | oropharynx | 0 | 11 | 0.00 |
| M | 59 | 4 | 2a | 0 | larynx | 1 | 93 | 0.01 |
| M | 67 | 2 | 2a | 0 | oropharynx | 1 | 77 | 0.01 |
| M | 53 | 4 | 2a | 0 | oral cavity | 1 | 68 | 0.01 |
| M | 44 | 2 | 1 | 0 | oropharynx | 1 | 53 | 0.02 |
| M | 52 | 4 | 1 | 0 | larynx | 1 | 48 | 0.02 |
| M | 54 | 2 | 1 | 0 | oral cavity | 1 | 47 | 0.02 |
| M | 55 | 4 | 2a | 0 | oral cavity | 1 | 43 | 0.02 |
| M | 54 | 2 | 2b | 0 | oral cavity | 2 | 59 | 0.03 |
| M | 66 | 2 | 1 | 0 | oral cavity | 1 | 29 | 0.03 |
| M | 68 | 4 | 1 | 0 | oral cavity | 1 | 29 | 0.03 |
| M | 39 | 3 | 2b | 0 | oral cavity | 2 | 49 | 0.04 |
| M | 52 | 1 | 3 | 0 | oropharynx | 2 | 48 | 0.04 |
| M | 42 | 4 | 2c | 0 | oral cavity | 5 | 113 | 0.04 |
| M | 50 | 2 | 2b | 0 | oral cavity | 2 | 41 | 0.05 |
| M | 39 | 2 | 1 | 0 | oral cavity | 1 | 19 | 0.05 |
| M | 46 | 4 | 1 | 0 | oral cavity | 1 | 19 | 0.05 |
| M | 56 | 2 | 1 | 0 | oral cavity | 1 | 19 | 0.05 |
| M | 41 | 4 | 2b | 0 | oral cavity | 2 | 37 | 0.05 |
| M | 51 | 2 | 2b | 0 | hypopharynx | 2 | 34 | 0.06 |
| M | 63 | 2 | 1 | 0 | oral cavity | 1 | 17 | 0.06 |
| M | 55 | 2 | 2b | 0 | oral cavity | 2 | 34 | 0.06 |
| M | 49 | 1 | 1 | 0 | oral cavity | 1 | 16 | 0.06 |
| M | 50 | 4 | 2c | 0 | hypopharynx | 3 | 45 | 0.07 |
| M | 42 | 4 | 2b | 0 | oral cavity | 2 | 29 | 0.07 |
| M | 54 | 3 | 2b | 0 | hypopharynx | 3 | 36 | 0.08 |
| M | 47 | 4 | 2b | 0 | oral cavity | 2 | 24 | 0.08 |
| M | 58 | 4 | 2b | 0 | oral cavity | 2 | 24 | 0.08 |
| F | 44 | 2 | 2b | 0 | oral cavity | 2 | 23 | 0.09 |
| M | 49 | 4 | 2c | 0 | oral cavity | 5 | 55 | 0.09 |
| M | 49 | 2 | 2b | 0 | oral cavity | 2 | 22 | 0.09 |
| F | 53 | 1 | 2b | 0 | oral cavity | 2 | 20 | 0.10 |
| M | 34 | 2 | 1 | 0 | oral cavity | 1 | 10 | 0.10 |
| M | 48 | 4 | 2b | 0 | hypopharynx | 3 | 28 | 0.11 |
| M | 57 | 4 | 2b | 0 | larynx | 5 | 45 | 0.11 |
| M | 52 | 3 | 2b | 0 | oral cavity | 3 | 27 | 0.11 |
| F | 62 | 1 | 2b | 0 | oral cavity | 2 | 17 | 0.12 |
| M | 55 | 1 | 2b | 0 | oral cavity | 2 | 16 | 0.13 |
| M | 44 | 1 | 2b | 0 | oropharynx | 4 | 29 | 0.14 |
| F | 49 | 1 | 2b | 0 | oral cavity | 4 | 28 | 0.14 |
| M | 51 | 2 | 2c | 0 | oral cavity | 6 | 41 | 0.15 |
| M | 38 | 4 | 2b | 0 | oral cavity | 4 | 27 | 0.15 |
| M | 58 | 3 | 2b | 0 | oral cavity | 5 | 33 | 0.15 |
| M | 56 | 1 | 1 | 0 | oral cavity | 1 | 6 | 0.17 |
| M | 45 | 2 | 2b | 0 | oral cavity | 3 | 18 | 0.17 |
| M | 58 | 2 | 2a | 0 | oral cavity | 1 | 6 | 0.17 |
| M | 61 | 4 | 2b | 0 | larynx | 5 | 29 | 0.17 |
| M | 53 | 3 | 2b | 0 | oral cavity | 3 | 17 | 0.18 |
| M | 43 | 2 | 2b | 0 | oral cavity | 2 | 11 | 0.18 |
| M | 51 | 2 | 2b | 0 | oral cavity | 2 | 10 | 0.20 |
| M | 56 | 4 | 2b | 0 | hypopharynx | 6 | 27 | 0.22 |
| M | 55 | 4 | 2b | 0 | oral cavity | 2 | 9 | 0.22 |
| M | 47 | 2 | 2b | 0 | oral cavity | 2 | 9 | 0.22 |
| M | 39 | 4 | 2b | 0 | oral cavity | 4 | 15 | 0.27 |
| M | 45 | 2 | 2b | 0 | oral cavity | 12 | 37 | 0.32 |
| M | 43 | 4 | 2b | 0 | oral cavity | 2 | 6 | 0.33 |
| M | 62 | 3 | 2b | 0 | oral cavity | 15 | 39 | 0.38 |
| M | 49 | 2 | 2b | 0 | oropharynx | 9 | 17 | 0.53 |
| F | 63 | 2 | 2b | 0 | oropharynx | 2 | 3 | 0.67 |
| M | 55 | 4 | 1 | 0 | oral cavity | 1 | 1 | 1.00 |
| M | 74 | 4 | 2b | 0 | oral cavity | 2 | 2 | 1.00 |
| M | 51 | 4 | 2b | 0 | oral cavity | 2 | 2 | 1.00 |
| F | 35 | 3 | 2a | 0 | oral cavity | 1 | 1 | 1.00 |
| M | 58 | 2 | 2a | 0 | oropharynx | 1 | 1 | 1.00 |
